# Feed tossing behaviour of Holstein cows: evaluation of physiological stress state and rumen fermentation function

**DOI:** 10.1186/s12917-022-03469-0

**Published:** 2022-10-17

**Authors:** Fuyu Sun, Qingyao Zhao, Xiaoyang Chen, Guangyong Zhao, Xianhong Gu

**Affiliations:** 1 State Key Laboratory of Animal Nutrition, Institute of Animal Science, Chinese Academy of Agricultural Sciences, No.2 Yuanmingyuan West Road, Beijing, China; 2 College of Animal Science and Technology, China Agricultural University, No.2 Yuanmingyuan West Road, Beijing, China

**Keywords:** Behaviour, Feed tossing, Holstein cow, Stress, Rumen fermentation, Immunity, Welfare

## Abstract

**Background:**

Abnormal or stereotyped behaviours in dairy cows are common in large-scale indoor farms and are usually accompanied by high physiological stress levels. Feed tossing is an abnormal behaviour commonly seen in cows while being fed, making farm management difficult. However, the reasons behind this behaviour have not been sufficiently reported. The objective of this study was to explore the changes in rumen fermentation, serum indicators, inflammatory conditions and the performance of cows with feed tossing behaviour. Holstein cows with similar lactation stages in the same barn were subjected to behaviour observations two times per day for 21 consecutive days. Ten cows with feed tossing behaviour (FT) and ten cows without abnormal behaviours (CON) were selected for further sampling. Plasma samples, rumen fluid, milk yield data of cows, and an indoor environment temperature-humidity index (THI) were collected.

**Results:**

There was no significant difference in average daily milk yield during the observation period between feed-tossing cows (*n* = 68) and the other cows (*n* = 112). The number of cows showing FT behaviour had a moderately strong negative linear correlation with the THI of the environment. Compared to the CON cows, the FT cows had higher cortisol, norepinephrine and urea nitrogen levels in plasma, as well as higher plasma levels of inflammatory indicators, including total protein, lactate dehydrogenase, albumin, aspartate aminotransferase levels, and the ratio of aspartate aminotransferase to alanine aminotransferase. The FT cows had no significant variations from the CON cows regarding their rumen fermentation indicators, such as pH, ammonia nitrogen, and volatile fatty acids. In addition, 16S rRNA analysis revealed that there might be no clear association between the diversity and abundance of rumen bacteria and feed tossing behaviour.

**Conclusions:**

Our findings suggested that cows might have suffered from high levels of physiological stress and immune state for a long period when they exhibited FT behaviour. The environmental THI could affect the FT behaviour of cows; as the THI increases, the willingness of cows to throw decreases. This work provided the first evidence that feed tossing might be a response associated with high levels of physiological stress and immune. It also explored our insights into a commonly observed behavioural response to cow welfare traits.

**Supplementary Information:**

The online version contains supplementary material available at 10.1186/s12917-022-03469-0.

## Introduction

Abnormal or stereotyped behaviours in dairy cows are of particular concern to farmers due to their complicated incidence patterns and difficulties in making appropriate diagnoses. Some abnormal or stereotyped behaviours, such as tongue rolling and crib biting, were reported to be associated with a prolonged state of physical stress, mental stress or changes in metabolism and inflammation in dairy cows [1–3]. Research on animal behaviour has been aiding in the development of solutions to improve dairy cattle welfare [4]. To date, researchers have reported several possible incentives for the development of abnormal or stereotyped behaviour in dairy cows: (1) Stress sensitivity and stress threshold [5]. When some individual cows are stressed, the stress level might quickly exceed their stress threshold, inducing their stereotyped behaviours in response to stress. (2) Genetic dispositions and behavioural tendencies. Some individuals tend to exhibit specific stereotypic behaviours during stress [6, 7]. (3) Individual growth experience and perception of stressors [8]. (4) Frustrated behavioural motivation [9]. (5) Low environmental richness and a lack of sufficient sensory stimulation. Cows in such environments tend to exhibit alternative behaviours as a way to escape the situation [10, 11]. (6) Specific roughage or minerals are short in the feed. When compared to calves given low-quality roughage (e.g., straw pellets), calves given high-quality roughage (e.g., hay) had less tongue rolling behaviour [12]. (7) Behavioural imitation and learning exist among individual animals [13]. The first three possible reasons are hereditary factors, while the remaining four are acquired or growth-related stimuli that lead to the formation of abnormal behaviours.

Feed tossing behaviour is a typical behaviour that occurs frequently throughout the seasons in the housing system for cows [14]. It often occurs in tie-up stalls or neck clamp confinement pens and occasionally in other husbandry systems. It is more prevalent when the feed is given from a bunk or trough whose base is not at floor level [15]. This behaviour manifests as follows: a mouthful of silage was picked up, flung upwards into the air by dipping the head and twisting the neck upwards, then partially fell on the animal's back. The feed tossing behaviour may have negative consequences for cattle management and the environment: (1) feed might be squandered, (2) the shed interior might be polluted, and (3) the slatted floor's function might be affected.

Although total mixed ration (TMR) diets provided in indoor faming can help cows swallow and ingest, it leads to a shortage of grass tearing and ripping activity (which would normally be performed 30,000 to 40,000 times per day when grazing on nature sward) [15]. Lack of these instinctive behaviours might increase the probability of stereotyped behaviours. Some researchers considered feed tossing behaviour a stereotyped behaviour based on certain traits [16], such as having no obvious function, being rhythmical and repetitive, and being used as a substitute for natural behaviour [17]. However, the tossing behaviour of dairy cattle has rarely been reported, particularly in relation to the parameters of the physiology condition and production performance of dairy cows.

This was a case‒control observational study with the hypothesis that physiological stress and rumen fermentation in cows with feed tossing behaviour are different from those in cows without feed tossing behaviour. To evaluate the relationship between feed tossing behaviour and target indicators, we observed 200 dairy cows and recorded their activity during feeding. We then selected typical cows with feed tossing behaviour (FT) or without feed tossing behaviour (CON) as our research targets. Samples from FT cows and CON cows were collected and analysed from multiple perspectives, including rumen nutritional metabolism, serum indicators and inflammatory conditions, environmental heat tolerance, and production performance. The results could help supplement the information on abnormal cow behaviours.

## Results

### Analysis of environment THI indicators and the feed tossing events

**Fig. 1:**
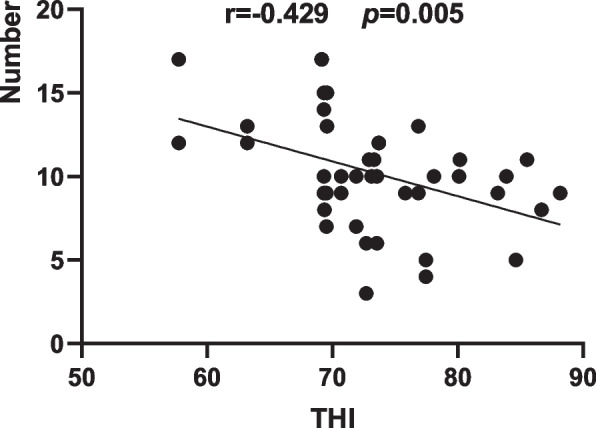
Correlation analyses between the environmental THI and the number of observed cows with feed tossing behaviour during the consecutive 21 days

The environmental THI recorded during the observation ranged from 68 to 88, corresponding to cows being in a state with no heat stress (THI < 72), mild heat stress (72 < THI < 78), and moderate heat stress (78 < THI < 88) [18]. Comparing the behaviour records with different THI intervals revealed a decreasing trend of feed tossing events with the increase in the degree of heat stress. Further study of the correlation between the number of tosses and THI values revealed a moderately strong negative correlation between the number of tosses and THI index (*r* = -0.429, *p* = 0.005, Fig. 1). The correlation indicates that the number of feed tosses was influenced by THI, and the higher the THI was, the lower the number of feed tosses.

### Analysis of ruminal fermentation and bacterial diversity

The rumen pH, NH3-N, and volatile fatty acid (VFA) concentrations are shown in Table 1. In the present study, there were no differences in rumen fermentation parameters.

**Table 1:** Fermentation parameters of rumen fluid sampled 2 h after feeding in dairy cow showing feed tossing behavior or no signs of abnormal behavior

Items	Experimental treatments^a^		SEM^b^	*P*-value
	FT	CON		
pH	6.275	6.373	0.053	0.354
Ammonia-N (mg/dL)	20.894	19.334	0.794	0.352
Acetate (mmol/L)	72.187	71.830	1.501	0.909
Propionate (mmol/L)	32.288	32.013	1.017	0.897
Butyrate (mmol/L)	0.760	0.795	0.037	0.652
Isobutyrate (mmol/L)	13.522	13.745	0.331	0.746
Valerate (mmol/L)	1.336	1.248	0.074	0.566
Isovalerate (mmol/L)	2.166	1.883	0.129	0.282
Total Volatile Fatty Acids (mmol/L)	122.260	121.513	2.698	0.894

^a^*FT* Feed tossing cows, *CON* Normal cows with no abnormal oral behavior

^b^*SEM* Standard error of the mean

**Fig. 2:**
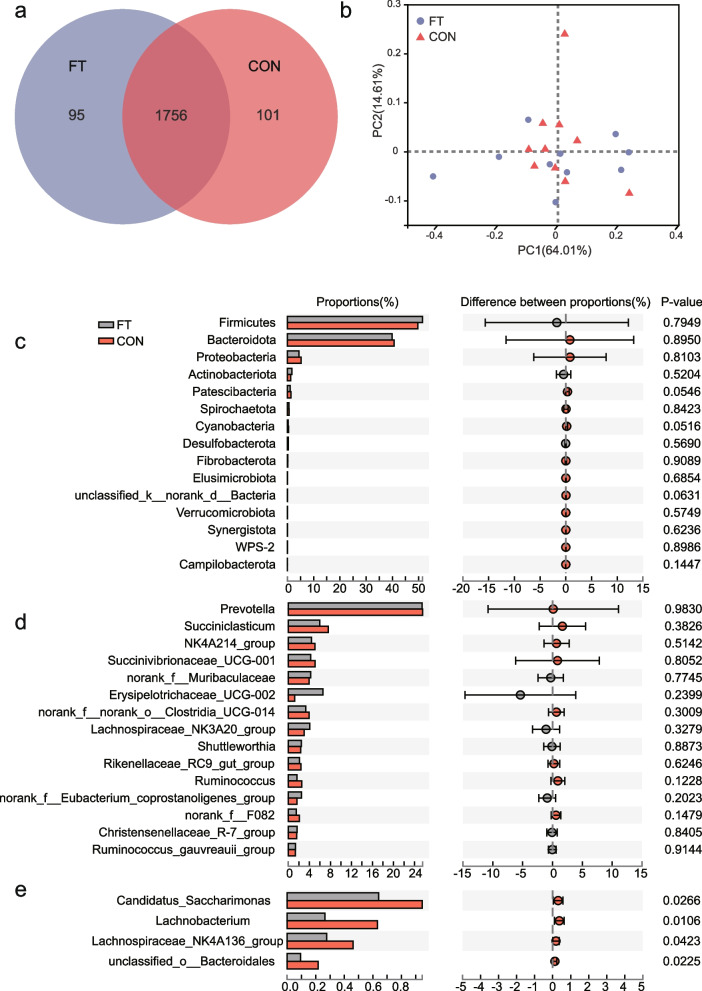
**a** Venn diagram of unique or shared OTUs in the FT (blue) and CON (red) groups. Ninety-five and 101 species were uniquely found in FT and CON cows, respectively, while 1756 species were found in both groups. **b** Principal component analysis (PCoA) of the ruminal bacterial community in the FT and CON groups. The first two principal components (PC1 and PC2) accounted for 78.62% of the total variation. **c-e** Top 15 abundant species at at the phylum level (**c**) and genus level (**d**) and species with significant differences between the FT and CON groups at the genus level (**e**). *P* values were calculated by Student’s t test. FT, cows with feed tossing behaviour; CON, cows without feed tossing behaviour

The diversity of rumen bacteria reflects the fermentation function of the rumen. We further analysed the ruminal fermentation changes with respect to rumen bacterial diversity. The total number of sequencing reads for all samples was between 30,000 and 46,000, with an average length of more than 430 nt. The current study identified 1,965 OTUs, 19 phyla, and more than 261 genera, and the Venn diagram depicted the unique or shared OTUs in the FT and CON groups (Fig. 2a). Supplementary Material 1 contains all taxonomic information.

Chao1 and Ace, Shannon and Simpson, Good's coverage indices were used to analyse the community richness, diversity, and sequencing authenticity, respectively [19]. Table 2 shows all these diversity indices, but they were not significantly different between the FT and CON groups.

**Table 2:** α diversity indicators of rumen bacteria of dairy cow showing feed tossing behavior or no signs of abnormal behavior

Items	Experimental treatments^a^		SEM^b^	*P-*value
	FT	CON		
Shannon	5.157	5.5033	0.613883	0.3075
Simpson	0.029636	0.019324	144.7131	0.4274
Ace^c^	1253.7	1383.2	46.79285	0.162
Chao1	1262.3	1397.1	158.1484	0.1405
Good’s Coverage	0.98931	0.98785	0.000311	0.1041

^a^*FT* Feed tossing cows, *CON*Normal cows with no abnormal oral behavior

^b^*SEM*, Standard error of the mean

^c^*ACE*, Abundance-based coverage estimator

Figure 2b shows that PCoA Axes 1 and 2 at the phylum level accounted for 64.10% and 14.61% of the total variation, respectively. However, based on the results, the bacterial community in the FT cows could not be separated from that in the CON cows by PCoA.

Differential analysis of ruminal bacteria at different levels was then conducted to investigate the effect of bacterial abundance on rumen fermentation. T test results at the genus level and phylum level (the species with the top 15 abundances) showed no significant differences in the abundance of bacteria in dairy cows (Fig 2c and d). Only four genera differed significantly, but in a low proportion (after the top 15 abundances, Fig. 2e). The bacterial abundance of *Candidatus Saccharimonas*, *Lachnobacterium*, *Lachnospiraceae NK4A136 group*, and *unclassified o-Bacteroidales* was significantly lower in the FT group of cows than in the CON group (*p* < 0.05).

### Analysis of plasma biochemistry and inflammatory indicators

Table 3 shows the differences in plasma stress indicators between the two groups. The COR (*p* = 0.004) and NE (*p* = 0.023) levels in FT cows were significantly higher than those in CON cows, and 5-HT (*p* = 0.085) also tended to increase. The other plasma stress indicators were not significantly different between groups.

**Table 3:** Differences on plasma stress indicators between two group cows showing feed tossing behavior or no signs of abnormal behavior

Items	Experimental treatments^a^		SEM^b^	*P*-value
	FT	CON		
Cortisol (ng/ml)	13.517	8.835	0.883	0.004
Heat shock protein 70 (pg/ml)	54.281	54.479	0.977	0.934
γ-hydroxybutyric acid (umol/L)	1.301	1.259	0.044	0.638
Epinephrine (ng/m)	3.130	3.190	0.047	0.530
Norepinephrine (ng/ml)	19.478	16.371	0.730	0.023
5-hydroxytryptamine (pg/ml)	574.262	480.434	28.139	0.085
Dopamine (nmol/L)	23.866	21.579	1.027	0.264

^a^*FT* Feed tossing cows, *CON* Normal cows with no abnormal oral behavior

^b^*SEM* Standard error of the mean

For plasma energy metabolic indicators, plasma T3, T4, GLU, TC, and LD in the FT cows did not differ from those in the CON cows significantly. The plasma BUN of the FT cows was significantly higher than that of CON cows (*p* = 0.026), and TG tended to decrease (*p* = 0.098, Table 4) in FT cows.

**Table 4:** Differences on plasma energy metabolic indicators between two group cows showing feed tossing behavior or no signs of abnormal behavior

Items	Experimental treatments^a^		SEM^b^	*P*-value
	FT	CON		
Thyroxine (ng/ml)	183.166	171.139	9.854	0.571
Triiodothyronine (ng/ml)	2.421	2.571	0.133	0.579
Glucose (mmol/L)	3.274	3.103	0.068	0.251
Blood urea nitrogen (mmol/L)	3.739	3.093	0.153	0.026
Triglycerides (mmol/L)	0.110	0.136	0.008	0.098
Total cholesterol (mmol/L)	6.833	6.400	0.278	0.407
Lactic acid (mmol/L)	1.190	1.283	0.074	0.537

^a^*FT* Feed tossing cows, *CON* Normal cows with no abnormal oral behavior

^b^*SEM* Standard error of the mean

For plasma immunity and inflammatory indicators, TP (*p* = 0.001), ALB (*p* = 0.017), AST (*p* = 0.028), and LDH (*p* = 0.019) in the FT cows showed different degrees of increase. The FT cows had a higher ratio of AST to ALT (*p* = 0.025). Moreover, IgA, IgG, IgM, GSH, NO, TNF-α, IL-6, IL-10, ALT, ALP, and CK were not significantly different between the two groups (Table 5).

**Table 5:** Differences on plasma immunity and inflammatory indicators between two group cows showing feed tossing behavior or no signs of abnormal behavior

Items	Experimental treatments^a^		SEM^b^	*P*-value
	FT	CON		
Immunoglobulin A (ug/ml)	109.399	110.793	2.737	0.807
Immunoglobulin G (mg/ml)	4.022	3.666	0.160	0.267
Immunoglobulin M (mg/ml)	1.101	0.990	0.048	0.250
Total protein (g/L)	68.670	64.110	0.756	0.001
Albumin (g/L)	41.520	37.540	0.887	0.017
Glutathione (ug/ml)	46.469	43.923	1.305	0.332
Nitric oxide (umol/L)	251.508	253.917	1.524	0.519
Tumor necrosis factor-α (ng/L)	264.256	239.665	11.557	0.286
Interleukin–6 (ng/L)	426.239	377.291	16.709	0.138
Interleukin–10 (pg/mL)	23.850	21.406	1.025	0.231
Aspartate aminotransferase (U/L)	126.889	87.333	8.332	0.028
Alanine aminotransferase (U/L)	38.300	36.300	1.019	0.395
Aspartate aminotransferase / Alanine aminotransferase	3.356	2.390	0.222	0.025
Alkaline phosphatase (U/L)	47.300	46.400	0.946	0.642
Lactate dehydrogenase (U/L)	213.700	168.400	10.186	0.019
Creatine kinas (U/L)	34.556	43.100	3.550	0.228

^a^*FT* Feed tossing cows, *CON* Normal cows with no abnormal oral behavior

^b^*SEM* Standard error of the mean

## Discussion

When fed a TMR, dairy cows have a natural tendency to continually sort through the feed and toss it forward where it is no longer within reach. The causes of feed tossing behaviour in dairy cows are the subject of numerous debates and hypotheses by farmers: 1. stress response caused by low and narrow feeding pen facilities or neck limitation; 2. poor feed palatability, inappropriate TMR processing, resulting in picky feed behaviour; 3. mosquito and fly bites on the back; 4. endogenous stress manifestation in cows; 5. playful behaviour.

### Rumen fermentation

A stable rumen fermentation performance is an important concern in dairy cattle production [20]. The TMR diet was designed as a homogenous mixture with the goal of minimizing the selective consumption of individual feed components by dairy cattle, promoting a steady-state condition conducive to continuous rumen function, and ensuring adequate intake of fibres [21]. However, dairy cattle have been demonstrated to preferentially select (sort) for the grain component and discriminate against the lengthier forage components, even when fed a TMR diet [22, 23]. Feed tossing, which might be a picking behaviour, occurred during feeding. Picky eating means that cows ate more concentrate and less forage diets, potentially affecting the stable rumen fermentation environment. Ruminal pH, NH3-N and VFAs, and bacterial flora abundance and diversity are the main indicators used to evaluate the rumen fermentation function [24, 25], and the stabilized ruminal microbial community is primarily important for the proper rumen environment [26]. According to our results, there were no significant differences in rumen pH, ammonia nitrogen, VFA indicators, or rumen bacterial alpha diversity between FT and CON cows. In both groups, bacteria from the Bacteroidetes and Firmicutes phyla still dominated the core microbiome, which was consistent with Golder's report [27]. The ratio of *Candidatus Saccharimonas*, *Lachnobacterium*, *Lachnospiraceae NK4A136 group*, and *unclassified o-Bacteroidales* in FT cows was significantly lower than that in CON cows. However, the four genera accounted for less than 1% of the total bacteria and had little impact on rumen fermentation. The above findings suggested that the cows' tossing behaviour may not result in significant changes in the fermentation pattern of the cows that are fed the conventional TMR.

DeVries et al. discovered that dairy cows sorting and picking TMR can result in the ration consumed by cows being higher than intended in fermentable carbohydrates and lower in effective fibre, increasing the risk of low rumen pH [28]. Higher intake of concentrate resulted in increased levels of VFA in the rumen [29]. However, as TMR diets in cattle farms are well established, the diets are cut to the appropriate length and mixed thoroughly [30]. The picking behaviour may not result in significant differences in the percentage of concentrate and coarse feed entering the rumen or other rumen indicators. This was consistent with the findings reported. Thus, this study could not determine whether the feed tossing behaviour was directly attributable to pick eating willingness. In the future, the post-feeding concentrate to coarse ratios of cows with feed tossing behaviour could be measured through a Pennsylvania sieve to learn more about this issue.

### Plasma physiology

The hormones COR and NE are commonly used to assess the stress level of animals [31, 32]. When cows are stressed, the secretion of hormones such as cortisol, epinephrine, and norepinephrine increases, causing significant changes in metabolism. On the one hand, this panel of hormones increases hepatic glycogenolysis and blood glucose levels, lipolysis and plasma free fatty acids. On the other hand, it leads to a reduction in protein anabolism and an increase in catabolism, as well as an increase in body protein turnover and nitrogen excretion. Furthermore, nitrogen deposition is reduced to meet the cow's needs for amino acid isomerization into glucose, acute phase protein synthesis, and other immune products [2], and then the immune system is activated. At present, there is no report regarding the role of stress, metabolic, and immunological index alterations in the blood of dairy cows with feed tossing behaviour. Abnormal or stereotypic behaviour in cows was correlated with high physiological stress and low levels of well-being [33]. Our findings were consistent with the above reports that the concentration of both COR and NE exhibits a significant increase in the plasma of dairy cattle with feed tossing behaviour. Plasma BUN levels were higher in the FT group, suggesting that the cows were in a state of increased protein mobilization and proteolytic metabolism. The individual sensitivity of cows to immune-mediated illnesses might be predicted by stress reactivity [34]. FT cows had significantly higher plasma TP and ALB levels and high levels of LDH and AST in regard to blood inflammatory indicators. This activation and mobilization of the immune system in the body were consistent with the trend described above for stress-related physiological changes in animals.

In cows in a stressed state, dynamic changes in a variety of parameters are required to maintain normal function. A phenotypic response developed by an animal to an individual stressor within the environment is acclimation behaviour to stress [33]. In this study, FT cows had higher levels of plasma indicators of physiological stress. Feed tossing behaviour might be a phenotypic response to certain environmental stressors. E M Schäfer reported that cows with feed tossing behaviour had significantly higher blink frequency (signs of arousal) than animals who did not toss feed, implying that feed tossing behaviour could be a way to release mental or physical stress or a type of playing behaviour [35]. However, it is still worth noting that the continued stress state would inevitably result in a decrease in cow body condition, changed milk anabolism, lower milk yield and milk quality, as well as an increased risk of disease in the production system.

### Environmental factors

In this study, we found that feed tossing events occurred centrally in the period following TMR feeding in the pen. Although some researchers considered that feed tossing behaviour might be related to repelling mosquitoes, a study from Purdue University, in which milking cows were watched for feed tossing behaviour for more than a year, found that tossing behaviour occurred not only in summer (when flies were concentrated) but also in winter and other seasons [14]. Therefore, the cause of tossing events may not be limited to the interference of mosquitoes and flies. In addition, when cows had flies on their backs, they usually repelled them by whipping tails. Through the video records of seven 24-h cameras installed across the barn trough, we did not observe frequent tail whipping behaviour in cows. As a result, it could be hypothesized that insects such as mosquitoes and flies caused less disturbance to dairy cows in the barn. However, it cannot be excluded as a triggering factor for the feed tossing behaviour.

In addition, the density of sweat glands in the skin of cows is lower than that of humans. When the environmental THI increases (THI > 68), the heat generated in the cow's body is dissipated by evaporation of sweat and conductive heat dissipation to maintain a constant body temperature [36]. Under high THI conditions (THI > 72), cows reflexively regulate the production of body heat to reduce the metabolic burden of releasing heat. Calorie-producing physiological processes such as milk production, food intake, and exercise will be inhibited by self-regulation. In addition, cows would increase their standing time [37] and spend less time lying and ingesting [38]. In this study, the number of cows expressing feed tossing behaviour during feeding decreased linearly as THI increased. This result indicated that heat stress reduced the willingness of cows to toss during feeding because this activity would additionally increase body heat. We speculated that feed tossing behaviour might be actively regulated by cows. In a high THI environment, the reduction in feed tossing behaviour was an adaptive adjustment of dairy cows to heat stress.

## Conclusion

Feed tossing behaviour in cows is often accompanied by high stress and changes in immune status. The feed tossing behaviour could be an acclimation sign to the variable stress status of some cows. The feed tossing behaviour does not significantly affect rumen fermentation function when TMR feeding is appropriately used. Further research into the effects of other factors, such as insects, on feed tossing behaviour should be conducted in well-controlled future studies.

## Materials and methods

### Animals and study design

This study was carried out at the Shandong Yinxiang Weiye Group Company in Cao County, Shandong Province, China (34°82′N, 115°54′E) in August 2020. Two hundred second-third parity lactating Holstein cows (days in milk = 136 ± 18, mean ± SD) in the same barn were included in this research. The barn for these 200 cows was concrete-floored without an outdoor area, where every cow had more than one bed. The cowshed was equipped with fans and sprinklers for cooling. Cows were fed a total mixed ration (TMR) diet three times daily at 08:30 am, 15:30 pm, and 20:30 pm, with free access to water and diet. Cows were milked three times daily at 08:00 am, 15:00 pm, and 20:00 pm. The rations were not changed during the experimental period. The dietary ingredients and nutritional content are described in Table 6. Faeces were removed by an automatic manure scraper system, and recycled manure solids were used as bedding for dairy cows. The bedding was replaced as needed, and the cow barn was disinfected thoroughly once a week to ensure hygienic cleanliness. The density of mosquitoes and flies in the barn was strictly controlled.

**Table 6:** Ingredients and nutrient composition of experimental diets (% dry matter basis)

Item	Value
Ingredients	Content, %
Alfalfa	10.39
Oat hay	2.42
Dandelion	0.48
Whole corn silage	48.33
Cottonseed	2.90
Beet pulp	2.42
Ground corn	7.49
Pressed corn	9.43
Soybean meal	8.70
Rapeseed meal	1.69
Distillers Dried Grains with Solubles	0.72
Extruded soybean	1.33
Mineral and vitamin mix ^a^	3.70
Nutrient composition	
Total dry matter	100.00
Crude Protein	17.06
Ether Extract	3.32
Neutral Detergent Fiber	35.75
Acid Detergent Fiber	18.20
Net Energy for Lactating Cow /(MJ/kg)	6.11

^a^Contained the following per kg of diets: VA 170 000 IU, VD 8 000 IU, VE 1 9000 IU, Ca 160 g, P 50 g, Fe 800 mg, Cu 680 mg, Mn 3 500 mg, Zn7 500 mg, Se 80 mg, I 400 mg, Co 38 mg

The temperature (T) and relative humidity (RH) were recorded every 20 min by an automatic temperature and humidity recorder (RC-4, China) during the testing days. THI was calculated based on averaged values (observation period) of T and RH by the formula: THI = (1.8 × T + 32) − [(0.55 − 0.0055 × RH) × (1.8 × T − 26.8)] (NRC, 1971). The daily THI value fluctuations during the sampling period varied similarly.

During the research period, the dairy cows were observed for 21 consecutive days, 2 times a day. Feed tossing behaviour was observed and recorded within half an hour of each feeding. After aggregating the results of 42 observations at 21 days, dairy cows expressing feed tossing behaviour for more than 14 days were classified as the FT group (*n* = 68), and cows without feed tossing behaviour were classified as the CON group (*n* = 112). Cows showing feed tossing behaviour for less than 14 days were classified as the potential mosquito and flies’ interference group and excluded (*n* = 20). Milk yield was recorded for 21 consecutive days in a parallel milking parlor, and the daily mean was calculated for each cow. Milk production, parity, and days of lactation in 68 FT and 112 CON cows were analysed by Student’s *t test*, showing no significant difference between the two groups (Table 7). Based on the results, 10 FT cows and 10 CON cows were matched using propensity score matching (PSM) and used for subsequent sampling. Potentially confounding factors (milk production, parity, and days of lactation age) were matched one-to-one using the nearest neighbour distance method with a caliber of 0.03.

**Table 7:** Feed tossing behavior observations of dairy cows and background information

Items	FT	CON^a^	SEM	*P-*value
Cow number	62	118	–	–
Milk yield^b^	41.041	41.55	1.282	0.798
Parity	2.30	2.53	0.091	0.220
Days in Milk^c^	167.71	178.56	9.018	0.623

^a^*FT* Feed tossing cows, *CON* Normal cows with no abnormal oral behavior

^b^Milk yield = Daily average of milk yield for 21 consecutive days during behavioral observations

^c^Average value during the consecutive 21 days

### Sampling

On the penultimate day of the study, blood samples (10 mL in each) were collected at 11:00 am (neither the feeding nor milking time) from the coccygeal vein using vacutainer tubes (BD Biosciences, San Jose, CA). Before sampling, the skin of the caudal vein of each cow was disinfected by a cotton applicator with alcohol. Plasma was separated through centrifugation at 3,000 × g for 10 min at 4 °C and stored at -20 °C for subsequent detection. Cortisol (COR), triiodothyronine (T3), and thyroxine (T4) contents were determined by radioimmunoassay (shine i2000, Beijing North Institute of Biotechnology Co., Ltd.). Glucose (GLU), lactate dehydrogenase (LDH), lactic acid (LD), alkaline phosphatase (ALP), alanine aminotransferase (ALT), aspartate aminotransferase (AST), creatine kinase (CK), triglyceride (TG), total cholesterol (TC), blood urea nitrogen (BUN), total protein (TP), albumin (ALB), and nitric oxide (NO) assays were conducted with an AU480 autoanalyzer (Olympus Co.). Dopamine (DA), 5-hydroxytryptamine (5-HT), epinephrine (E), norepinephrine (NE), γ-hydroxybutyric acid (GABA), glutathione (GSH), heat shock protein 70 (HSP-70), immunoglobulin A (IgA), immunoglobulin G (IgG), immunoglobulin M (IgM), interleukin–6 (IL-6), interleukin–10 (IL-10), and tumour necrosis factor-α (TNF-α) were measured following the manufacturer's instructions by commercial ELISA kits. All colorimetric data were measured with the Thermo Multiskan Ascent (Waltham, MA, USA).

On the last day, rumen fluid samples were collected by an oral stomach tube sampler 2 h after morning feeding and strained through 4 layers of cheesecloth to obtain rumen fluid. The first 50 ml of rumen fluid of each cow was discarded to reduce contamination with saliva. Ruminal pH was immediately measured using a pH metre (PB-10, Sartorius, Germany). A 20 mL rumen fluid sample was acidified after mixing with 2 mL of 25% metaphosphoric acid and stored at − 80 °C for volatile fatty acid (VFA) analysis. Individual and total VFAs were separated and quantified by gas chromatography (GC-2010, Shimadzu, Kyoto). A 20 mL rumen fluid sample was processed to analyse the ammonia-N (NH3-N) levels after mixing with 0.4 mL of 50% sulfuric acid and stored at − 20 °C. The NH3-N concentration was determined by the indophenol method. The remaining part of the rumen fluid was stored at − 80 °C for bacterial analysis.

### Bacterial communities

Bacterial analysis of rumen fluid samples was carried out using high-throughput 16S rRNA gene sequence technology, as described in a previous study [3]. UPARSE (version 7.1, http://drive5.com/uparse/) was used to cluster the operational taxonomic units (OTUs, sequences with similarity ≥ 97%). Phylotype taxonomic information was assigned using the RDP classifier with a confidence threshold of 0.7 trained on the 16S rRNA Greengenes database (http://greengenes.lbl.gov) for bacteria. Alpha diversity was quantified using the Shannon and Simpson (to estimate community diversity), Ace and Chao1 (to estimate community richness) indices with QIIME2 (https://docs.qiime2.org/2019.7/tutorials/overview/). Beta diversity was assessed by principal coordinate analysis (PCoA) based on unweighted UniFrac distance metric analysis. The Stats package in R language (https://github.com/microbiota) was used for visualizing diversity analysis. The PCoA figure was used to show overall similarities in the structure of the bacterial community among the samples.

### Statistical analysis

Before analysis, we discarded the variables that showed a significant skew pattern by the normal distribution test for all measured variants. All data are reported as least-squares means ± SEMs. Student’s *t test* was applied to analyse the differences in rumen pH, VFAs, NH3-N, and plasma parameters between the FT and CON groups. QIIME2 (version 2019.07) and R (R Core Team, 2020) were used to examine bacterial sequencing profiles. First, the differences in diversity measures between groups were assessed using a nonparametric Kruskal‒Wallis test (QIIME2 software; version 2019.07). A distance-based (Bray‒Curtis distances) PERMANOVA in QIIME2 was also used to determine whether bacterial composition differed between groups. To assess whether bacterial groups were differentially numerous between FT and CON cows, analysis of microbiome composition tests was performed by R (R Core Team, 2020) at the phylum and genus levels. The differential taxa were calculated using Benjamini‒Hochberg correction at a 5% level of significance to account for false discoveries. A Wilcoxon rank sum test was used to analyse intergroup differences for taxa that differed significantly across groups as indicated by the analysis of microbiome composition test [39]. For all tests, values with *p* < 0.05 were regarded as statistically significant, and 0.05 < *p* ≤ 0.10 were regarded as trends.

## Supplementary Information

**Additional file 1:** **Supplementary Material 1.** Taxonomic information of the 16S rRNA sequences. Domain, Kindom, Phylum, Class, Order, Family, Genus, Species: the taxonomic units of bacteria. OTU, the operational taxonomic unit. FT, cows with feed tossing behavior; CON, cows without abnormal behavior.

## Data Availability

The 16S rRNA sequence datasets generated or analysed during the current study are available in the NCBI Sequence Read Archive database (https://www.ncbi.nlm.nih.gov/sra/) under project no. PRJNA690677.
