# Supplementary material for: Feed tossing behaviour of Holstein cows: evaluation of physiological stress state and rumen fermentation function
Source: BMC Vet Res. 2022 Oct 17;18:371. doi: 10.1186/s12917-022-03469-0 (PMC9575279; doi:10.1186/s12917-022-03469-0)
Supplement: Supplementary file 1 — Additional file 1: Supplementary Material 1. Taxonomic information of the 16S rRNA sequences. Domain, Kindom, Phylum, Class, Order, Family, Genus, Species: the taxonomic units of bacteria. OTU, the operational taxonomic unit. FT, cows with feed tossing behavior; CON, cows without abnormal behavior. [file 12917_2022_3469_MOESM1_ESM.zip › Description of Supplementary Material 1.docx]

**Description of Supplementary Files**

**File Name**: Supplementary Material 1

**Description**: Taxonomic information of the 16S rRNA sequences. Domain, Kindom, Phylum, Class, Order, Family, Genus, Species: the taxonomic units of bacteria. OTU, the operational taxonomic unit. FT, cows with feed tossing behavior; CON, cows without abnormal behavior.
